# *QuickStats:* Sepsis-Related* Death Rates^†^ Among Persons Aged ≥65 Years, by Age Group and Sex — National Vital Statistics System, United States, 2021

**DOI:** 10.15585/mmwr.mm7238a5

**Published:** 2023-09-22

**Figure:**
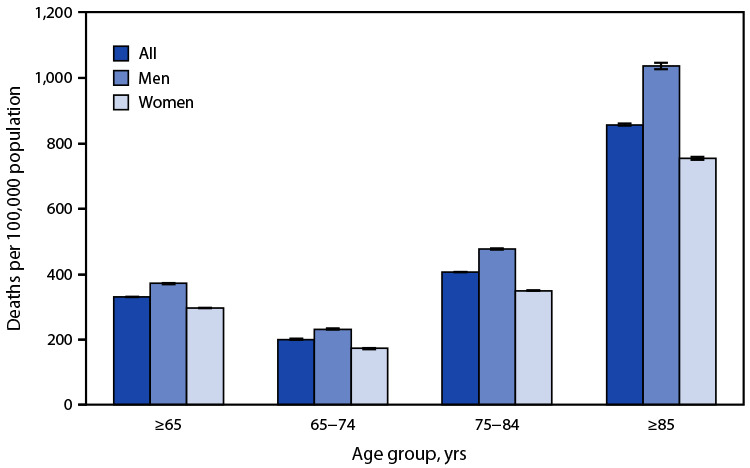
In 2021, the sepsis-related death rate among persons aged ≥65 years was 330.9 deaths per 100,000 population; the rate among men (371.7) was higher than that among women (297.4). Sepsis-related death rates among men were higher than those among women in each age group: 232.7 versus 173.0 (65–74 years), 477.3 versus 349.8 (75–84 years), and 1,037.8 versus 755.5 (≥85 years). Sepsis-related death rates increased with age from 201.1 among persons aged 65–74 years to 858.3 among those aged ≥85 years. Sepsis-related death rates increased with age among both men and women.

**Source:** National Center for Health Statistics, National Vital Statistics System, Mortality Data, 2021. https://wonder.cdc.gov/mcd.html

For more information on this topic, CDC recommends the following link: https://www.cdc.gov/sepsis/

